# Optimizing individualized treatment strategy based on breast cancer organoid model

**DOI:** 10.1002/ctm2.380

**Published:** 2021-03-31

**Authors:** Bo Pan, Xuelu Li, Dongyi Zhao, Ning Li, Kainan Wang, Man Li, Zuowei Zhao

**Affiliations:** ^1^ Department of Oncology and Department of Breast Surgery The Second Hospital of Dalian Medical University Dalian China; ^2^ Department of Foreign Language Dalian Medical University Dalian China

Dear Editor,

Breast cancer (BC) is one of the most common cancers and the cause of cancer death world‐wide among females.^1^ BC has profound genetic heterogeneity and tumor heterogeneity, which is closely associated with poor prognosis and survival, leading determinants of therapeutic resistance and treatment failure.^2^, ^3^ Therefore, an accurate assessment of tumor pharmacological heterogeneity is required for the development of effective therapies. A great number of studies have been done using cancer cell lines and patient‐derived xenografts, which include different advantages. However, multiple shortcomings are also present in clinical application.^4^, ^5^ Three‐dimensional organoid culture models provide us opportunities to conduct clinical and translational cancer research.^6^ The organoids can be derived from cancer patient materials of various tumor tissues.^6^, ^7^ They recapitulate tumor heterogeneity of the original tumor tissue, predicting the drug response of individualized patients.^8^ Recently, we have successfully established patient‐derived tumor organoids (PDTOs) from the uncommon pathological types of papillary carcinoma and mammary Paget's disease.^9^, ^10^ To study molecular pathogenesis of more Chinese population BC patients, we seek to establish a larger organoid platform. To the best of our knowledge, no Chinese population‐derived BC organoids have been established in China yet.

In this study, we obtained 48 human BC samples from primary tumors, liver metastases, and metastatic axillary lymph nodes in 40 BC patients for PDTOs generation. Tumor tissue specimens were processed by anatomical shredding, collagen digestion, lysing red blood cells, etc. Then the tumor cells were embedded in an extracellular matrix to form organoids. Ultimately, we successfully generated 35 PDTOs from 29 BC patients that were cultured for at least 6 months (Figures 1, S1, and S2). The successful rate of the establishment from BC patients was up to 72.5% (29/40), and the organoid establishment from tumor specimens was 72.9 % (35/48). Based on the location of breast lesions, we established 22 unilateral BC PDTOs, six multifocal BC PDTOs and six bilateral BC PDTOs. According to the molecular classification of BC, we established 23 Luminal BC PDTOs, nine HER2‐positive BC PDTOs, and three triple negative (TN) BC PDTOs. We found the organoids from BC patients with the following factors, including younger than 54 years old, Luminal classification, pTNM stage N1, and Vascular invasion, were more likely to be established successfully (Figure 1B). The median time for establishing PDTOs is 28 (95% CI 19∼43) days which could be used to make clinical decisions in a timely fashion (Figure S3). The morphology of these organoids was either thin‐walled cystic or compact without a cavity (Figure 1A). The PDTOs were cultured for long‐term expansion over 6 months without change in organoid morphology and maintained proliferative activity (Figure 1C). The PDTOs were reconstituted successfully, and the spherical organoid morphology remained the same when they underwent cryopreservation after resuscitation (Figure 1D).

**FIGURE 1:**
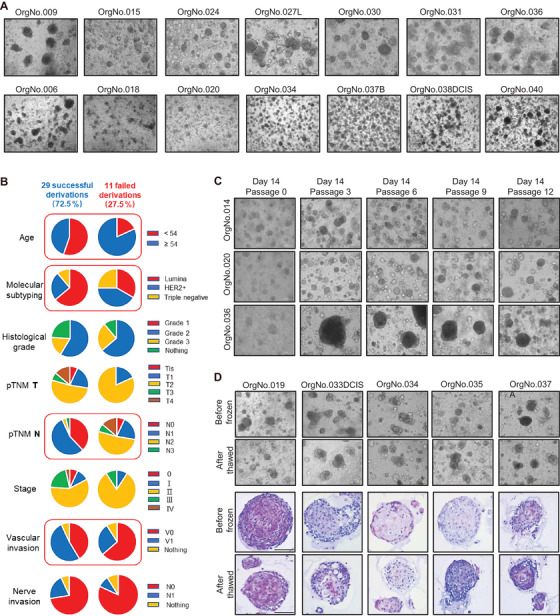
Establish Chinese population‐derived BC organoids. (A) Bright‐field microscopy images of organoids from different BC patients. Scale bar, 200 μm. (B) The pie charts show the outcome of attempts to derive organoids from 40 individual BC patients. BC organoids from 29 patients were successfully derived. For the 11 failed derivations, the points of failure are shown. Demographics from each group are displayed, including age, molecular subtyping, histological grade, pTNM T, pTNM N, stage, vascular invasion, nerve invasion. (C) Bright‐field microscopy images of PDTOs cultured for 2 weeks. Scale bar, 200 μm. (D) Bright‐field microscopy images of PDTOs before freezing and after thawing. Scale bar, 200 μm. H&E staining images of PDTOs before freezing and after thawing. Scale bar, 100 μm

Next, we observed that the phenotypes of PDTOs were consistent with the histological characteristics of BC (Figure 2). The histopathological high‐fidelity phenomenon was observed among bilateral BC PDTOs, multifocal BC PDTOs, and their original BC tissues (Figure 2). The organoids also retained the expression level of ER, PR, HER2, and Ki‐67 as similar as original BC (Figure 2). We performed whole genome sequencing with organoids derived from three neoadjuvant BC patients and observed genomic fidelity of the organoids (Figures S4 and S5).

**FIGURE 2:**
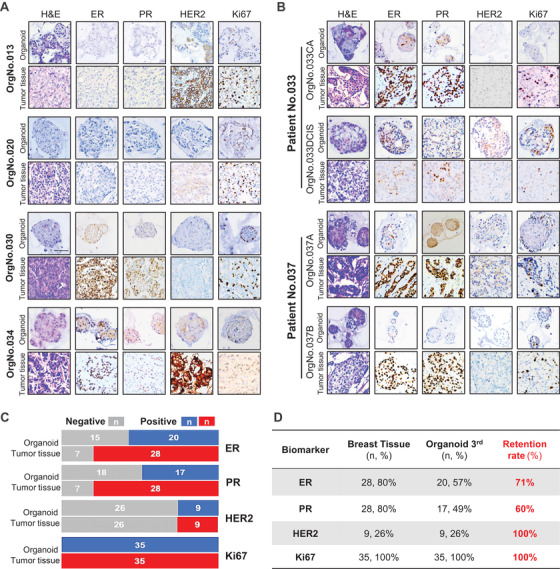
PDTOs recapitulate the pathological characteristics of the original breast tissues. H&E‐stained and IHC‐stained images of PDTOs and (A) their original unilateral BC tissues, (B) their original multifocal BC tissues. Scale bars, 100 μm. (C‐D) Stacked bar chart and table showing the number and percentage of breast cancer and organoids that are receptor positive (organoid: blue, tumor tissue: red) and negative (gray) grouped per original tumor receptor status

Additionally, we studied interpatient and intrapatient drug response heterogeneity of PDTOs to commonly used clinical drugs. To investigate interpatient drug response heterogeneity, we applied eight clinically used adjuvant therapy drugs (Tamoxifen, Fulvestrant, Paclitaxel, Docetaxel, Cyclophosphamide, Epirubicin, Carboplatin, Palbociclib) to perform drug sensitivity tests on eight PDTOs from different molecular types of BC patients. The drug response of Luminal type BC PDTOs to various drug treatments, especially endocrine therapy, was better than that of the other two molecular subtypes (Figures 3A and S6). The HER2‐positive BC PDTOs and TN BC PDTOs were less sensitive to endocrine drugs and more sensitive to chemotherapy drugs (Figures 3A and S6). Then, we investigated intrapatient drug response heterogeneity. We performed drug sensitivity tests on the bilateral and multifocal BC PDTOs, including six bilateral BC PDTOs from three bilateral BC patients and six multifocal BC PDTOs from three multifocal BC patients (Figures 3B, 3C, S6, S7, and S9). Altogether, we found that PDTOs derived from individual BC patients exhibited inter‐ and intrapatient drug response heterogeneity.

**FIGURE 3:**
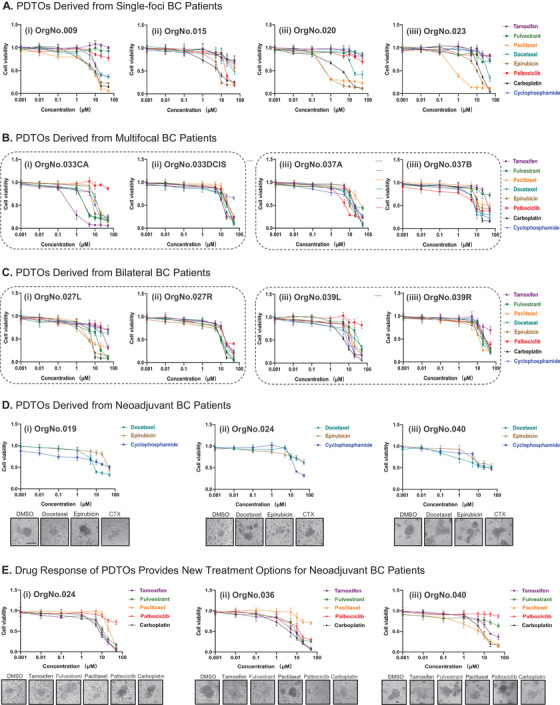
Pharmacological characteristics of PDTOs derived from different BC patients. (A) Drug dose‐response curves of PDTOs from different molecular types of BC patients for Tamoxifen, Fulvestrant, Paclitaxel, Docetaxel, Cyclophosphamide, Epirubicin, Carboplatin, Palbociclib. Mean ± SD of results from three independent experiments is shown for each drug. (B) Drug dose‐response curves of multifocal BC PDTOs for Tamoxifen, Fulvestrant, Paclitaxel, Docetaxel, Cyclophosphamide, Epirubicin, Carboplatin, and Palbociclib. Mean ± SD of results from three independent experiments is shown for each drug. (C) Drug dose‐response curves of bilateral BC PDTOs for Tamoxifen, Fulvestrant, Paclitaxel, Docetaxel, Cyclophosphamide, Epirubicin, Carboplatin, and Palbociclib. Mean ± SD of results from three independent experiments is shown for each drug. (D) Drug dose‐response curves of neoadjuvant BC PDTOs for Docetaxel, Cyclophosphamide, and Epirubicin. Mean ± SD of results from three independent experiments is shown for each drug. Bright‐field microscopy images of neoadjuvant BC PDTOs were treated with indicated drugs. Scale bars, 200 μm. CTX: Cyclophosphamide. (E) Drug dose‐response curves of neoadjuvant BC PDTOs for Tamoxifen, Fulvestrant, Paclitaxel, Palbociclib, and Carboplatin. Mean ± SD of results from three independent experiments is shown for each drug. Bright‐field microscopy images of neoadjuvant BC PDTOs were treated with indicated drugs. Scale bars, 200 μm

At last, we systematically assessed whether the in vitro drug response of PDTOs was correlated with BC patients’ clinical response to neoadjuvant therapy (Figures 3D, S8, and S10). We chose five neoadjuvant BC PDTOs derived from BC patients undergoing surgery after neoadjuvant chemotherapy. All five BC patients were given neoadjuvant therapy under TEC program (Docetaxel, Cyclophosphamide, Epirubicin), and their clinical response to neoadjuvant therapy was poor with residual lesions (Figure S10). The neoadjuvant BC PDTOs were exposed to Docetaxel, Cyclophosphamide, Epirubicin treatment in vitro. Retrospectively, we compared the drug response of neoadjuvant BC PDTOs with the neoadjuvant BC patients’ clinical response. Five neoadjuvant BC PDTOs were not sensitive to 1∼2 neoadjuvant chemotherapy drugs that was correlated to neoadjuvant patients’ clinical response (Figures 3D and S8). To provide novel treatment options for non‐pathological complete response BC patients, we performed drug screening to determine the effects of other drugs (Tamoxifen, Fulvestrant, Paclitaxel, Palbociclib, Carboplatin) on neoadjuvant BC PDTOs, and they showed distinct degrees of sensitivity to different drugs (Figures 3D and S8). Indeed, neoadjuvant BC PDTOs could provide novel adjuvant therapeutic options for neoadjuvant BC patients.

In conclusion, Chinese population‐derived BC organoids model could largely retain histological and genomic characteristics of original BC tissues which would provide a valuable preclinical model system to guide individualized treatment choice in the clinic (Figure S11).

## FUNDING INFORMATION

This work was supported by the National Natural Science Foundation of China (grant numbers: 82072934 and 81673762 to Zuowei Zhao and 81872156 to Man Li), Provincial Foundation of Liaoning (grant numbers: LR2017012 to Zuowei Zhao and 2019‐BS‐072 to Xuelu Li), and Innovation Foundation of Dalian (grant number: 2018J11CY026 to Zuowei Zhao).

## CONFLICT OF INTEREST

The authors declare that they have no competing interests.

## ETHICS APPROVAL AND CONSENT TO PARTICIPATE

Tumor specimens were obtained with the approval of the ethics committee of The Second Hospital of Dalian Medical University. Written, informed consent was obtained from patients.

## AUTHOR CONTRIBUTIONS

Bo Pan, Xuelu Li, Dongyi Zhao, Ning Li, and Kainan Wang performed experiments and analysis and prepared the manuscript. Bo Pan, Xuelu Li, Man Li, and Zuowei Zhao conceived and designed the experiments. All authors read and approved the final manuscript.

## AVAILABILITY OF DATA AND MATERIALS

All data during this research are included in this published article.

## Supporting information

Figure S1. Clinicopathologic characteristics of 29 BC patients whose organoids were successfully established.

Figure S2. The characteristics of breast cancer organoids.

Figure S3. The time interval from specimen collection to the establishment of the third‐generation organoids.

Figure S4. Genomic Characterization of the organoids derived from neoadjuvant BC patients.

Figure S5. Genomic Characterization of the organoids derived from neoadjuvant BC patients.

Figure S6. Pharmacological characteristics of PDTOs derived from different BC patients.

Figure S7. Bright‐field microscopy images of in vitro drug responses of BC PDTOs to several clinically used drugs.

Figure S8. PDTOs drug response correlates with clinical drug resistant response of original neoadjuvant BC patients and provides new treatment options for neoadjuvant BC patients.

Figure S9. Clinical imaging characteristics of bilateral BC patients and multifocal BC patients.

Figure S10. Clinical imaging characteristics of tumor changes before and after neoadjuvant therapy in BC patients.

Figure S11. PDTOs‐based co‐clinical trials in breast cancers.
