# Supplementary material for: Optimizing individualized treatment strategy based on breast cancer organoid model
Source: Clin Transl Med. 2021 Mar 31;11(4):e380. doi: 10.1002/ctm2.380 (PMC8012563; doi:10.1002/ctm2.380)
Supplement: Supplementary file 1 — Figure S1. Clinicopathologic characteristics of 29 BC patients whose organoids were successfully established. [file CTM2-11-e380-s009.pdf]

Figure S1

Variables	No. of Patients (%) (N=29)
**Age at BC Diagnosis**	
Median (range)	51 (31-74)
**Lesion type**	
Unilateral BC	22 (75.9%)
Bilateral BC	3 (10.3%)
Multifocal BC	3 (10.3%)
BC liver metastasis	1 (3.4%)
**Receptor Subtype**	
Luminal	18 (62.1%)
HER2+	7 (24.1%)
Triple negative	3 (10.3%)
Triple negative + HER2+ (Bilateral BC)	1 (3.4%)
**Histological Grade**	
Grade 1	0
Grade 2	17 (58.6%)
Grade 3	5 (17.2%)
Nothing	7 (24.1%)
**pTNM T**	
Tis	2 (6.9%)
T1	6 (20.7%)
T2	15 (51.7%)
T3	2 (6.9%)
T4	4 (13.8%)
**pTNM N**	
N0	11 (37.9%)
N1	16 (55.2%)
N2	1 (3.4%)
N3	1 (3.4%)
**pTNM M**	
M0	28 (96.6%)
M1	1 (3.4%)
**Clinical Stage**	
0	2 (6.9%)
Ⅰ	3 (10.3%)
Ⅱ	17 (58.6%)
Ⅲ	6 (20.7%)
Ⅳ	1 (3.4%)
**Vascular invasion (Patients)**	
V0	12 (41.4%)
V1	15 (51.7%)
Nothing	2 (6.9%)
**Nerve invasion**	
N0	21 (72.4%)
N1	6 (20.7%)
Nothing	2 (6.9%)
**Preoperative treatment**	
Neoadjuvant therapy	6 (20.7%)
Without neoadjuvant therapy	23 (79.3%)
