# Supplementary figures and images for: Optimizing individualized treatment strategy based on breast cancer organoid model
Source: Clin Transl Med. 2021 Mar 31;11(4):e380. doi: 10.1002/ctm2.380 (PMC8012563; doi:10.1002/ctm2.380)

Figure S2

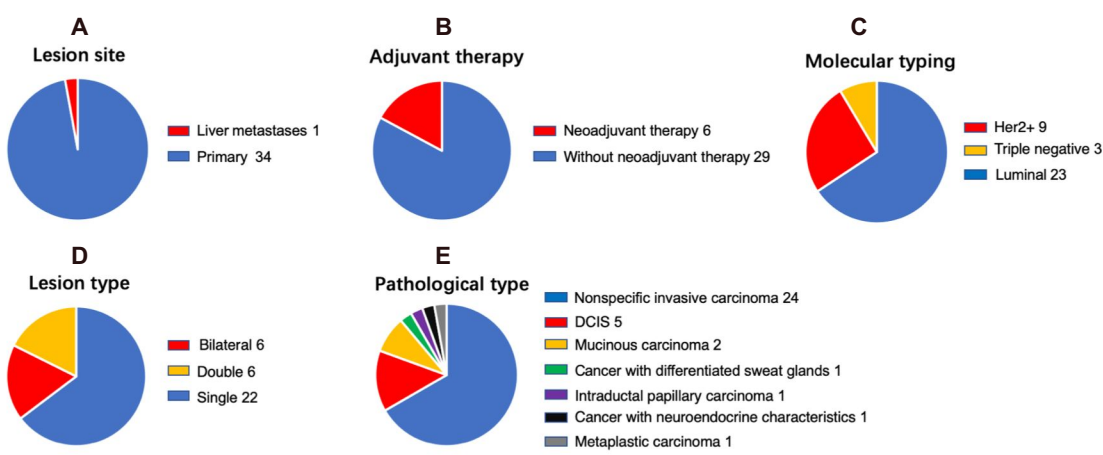

Supplement: Supplementary file 2 — Figure S2. The characteristics of breast cancer organoids. [file CTM2-11-e380-s007.pdf]

Figure S3

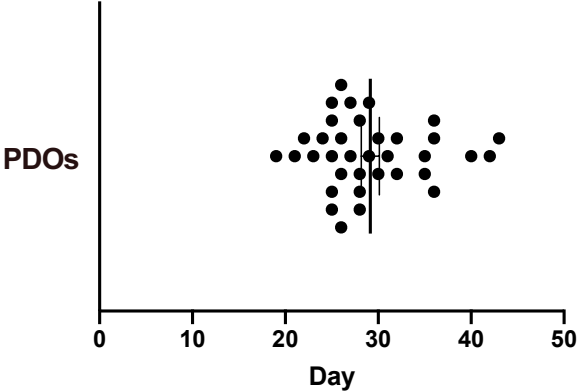

Supplement: Supplementary file 3 — Figure S3. The time interval from specimen collection to the establishment of the third‐generation organoids. [file CTM2-11-e380-s003.pdf]

Figure S4

A

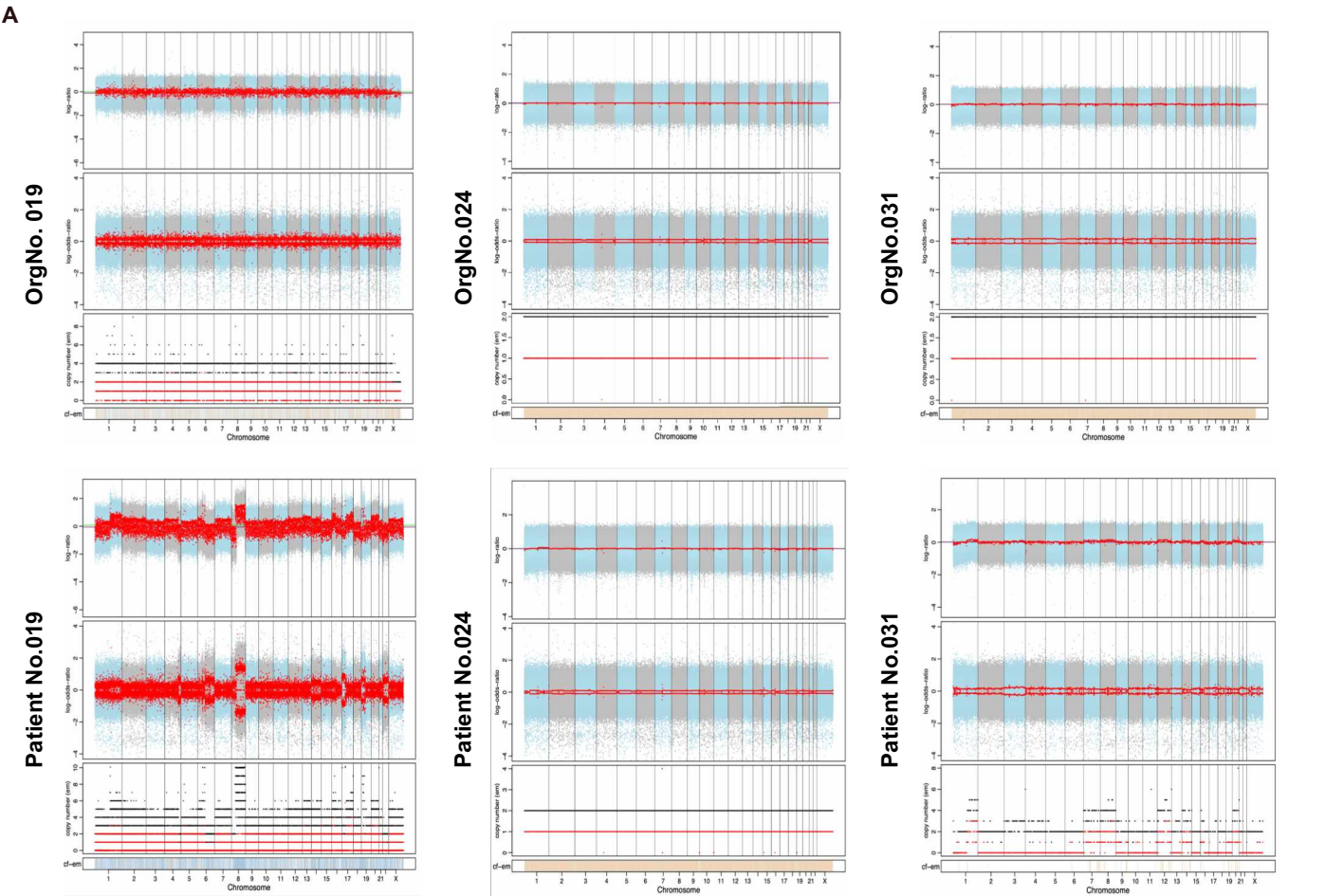

B

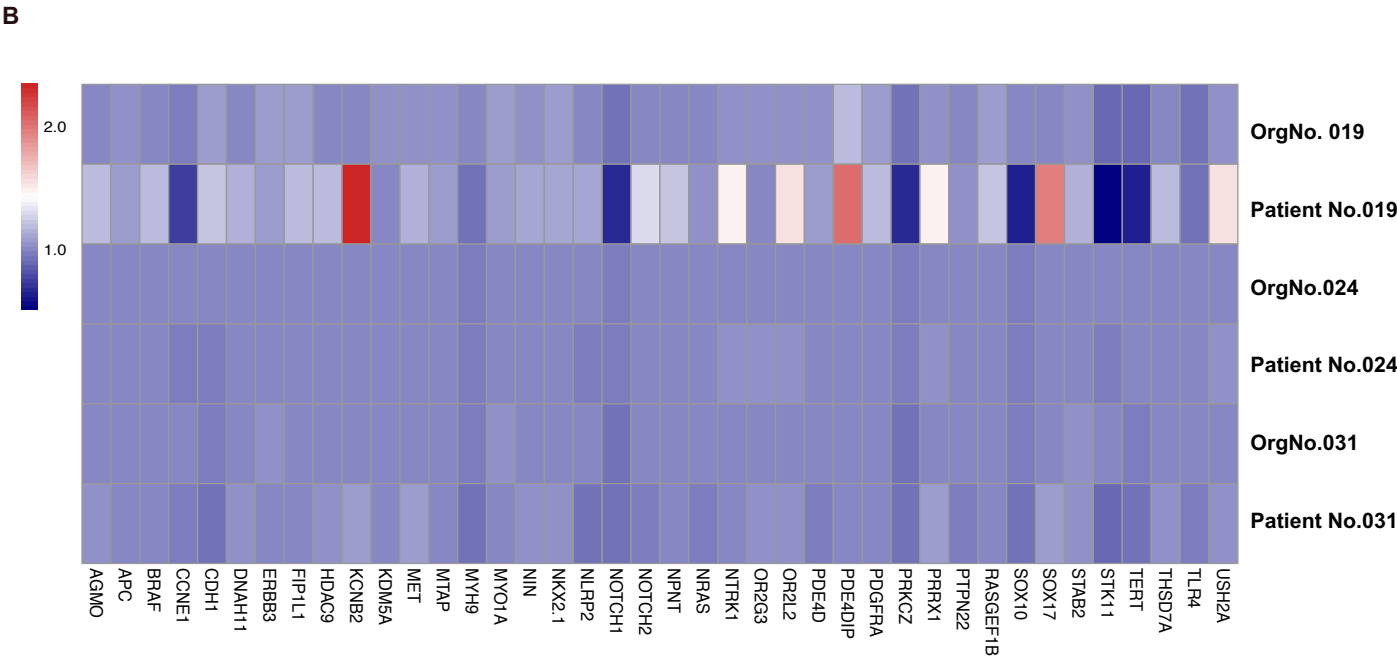

Supplement: Supplementary file 4 — Figure S4. Genomic Characterization of the organoids derived from neoadjuvant BC patients. [file CTM2-11-e380-s011.pdf]

Figure S5

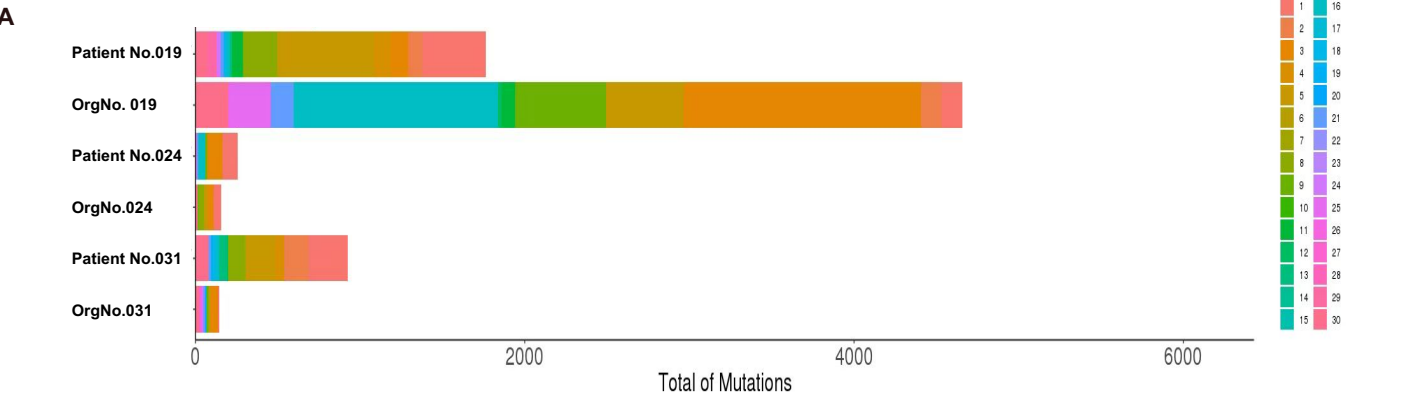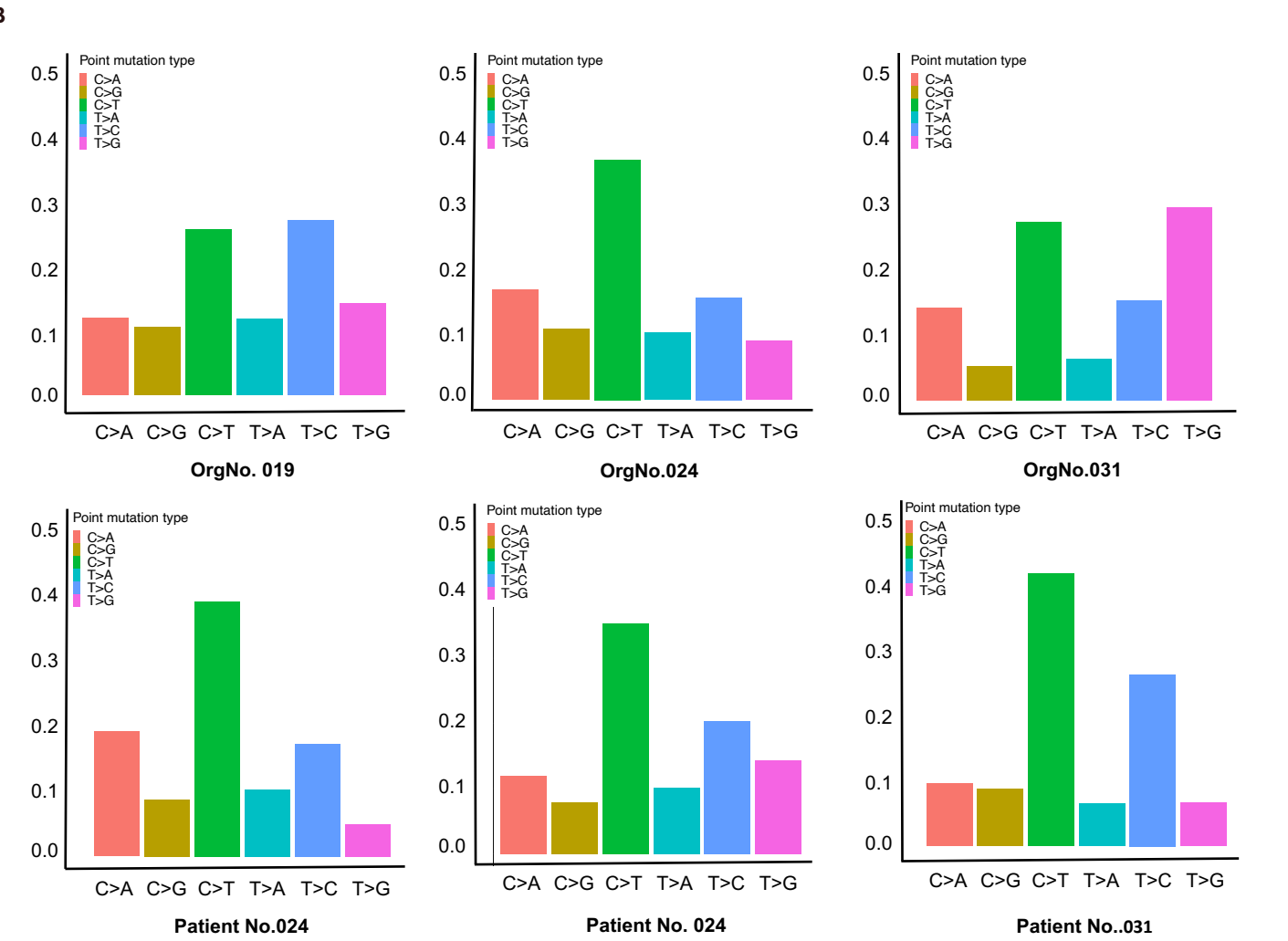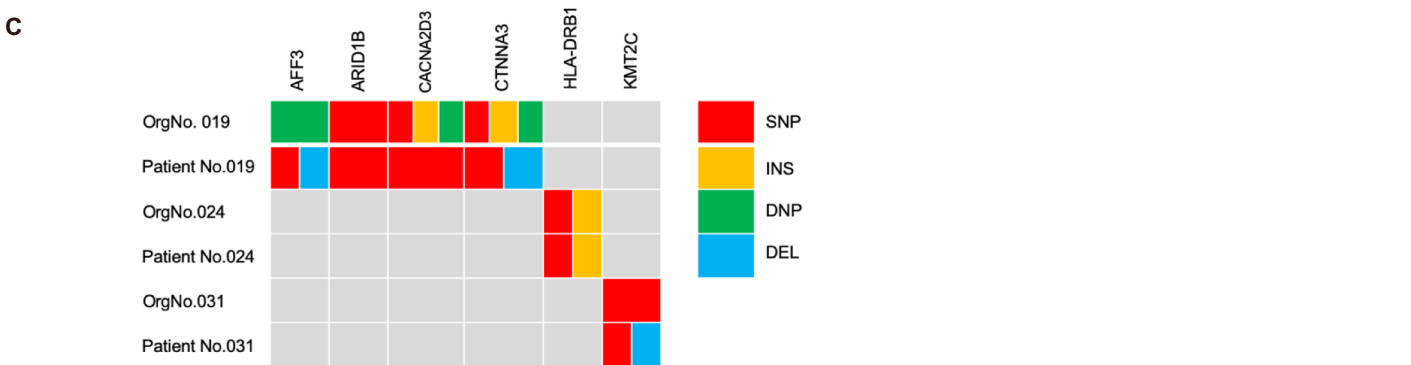

Supplement: Supplementary file 5 — Figure S5. Genomic Characterization of the organoids derived from neoadjuvant BC patients. [file CTM2-11-e380-s005.pdf]

Figure S7

A

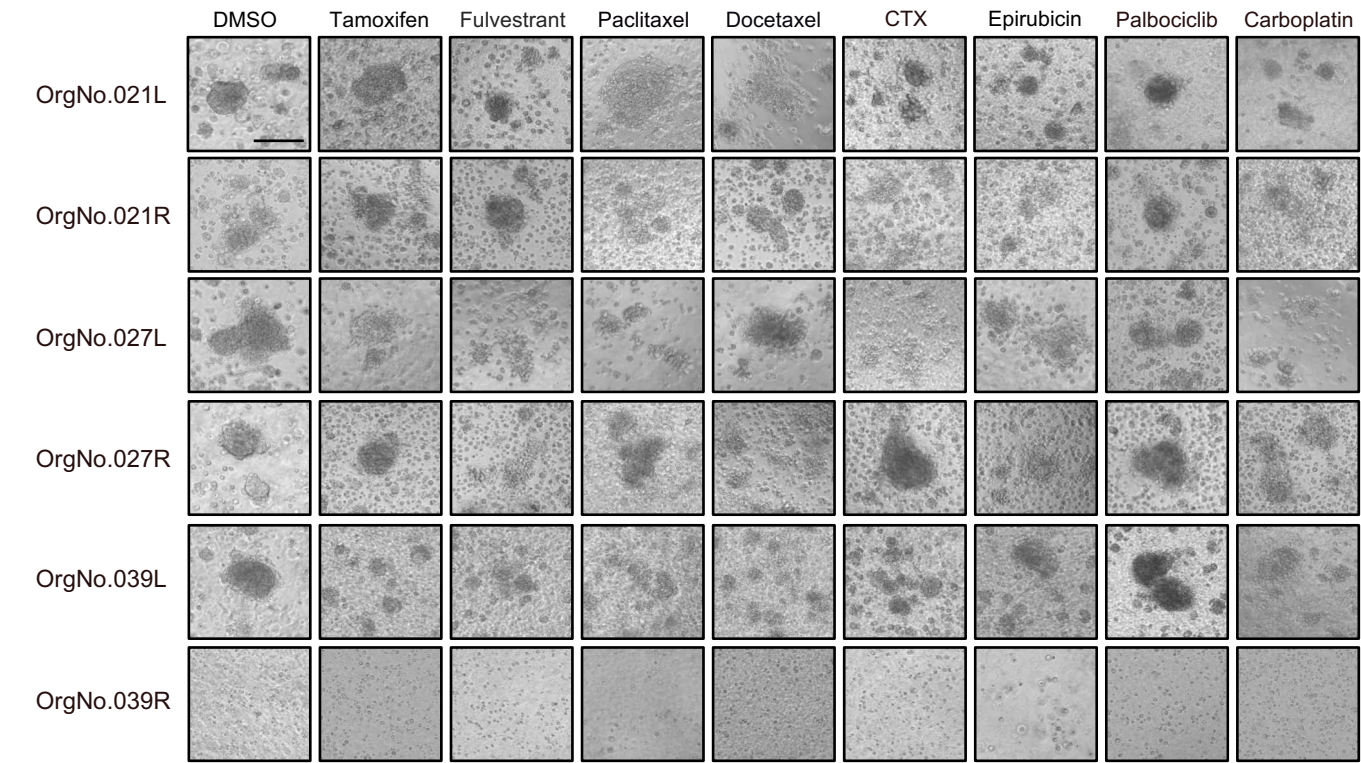

B

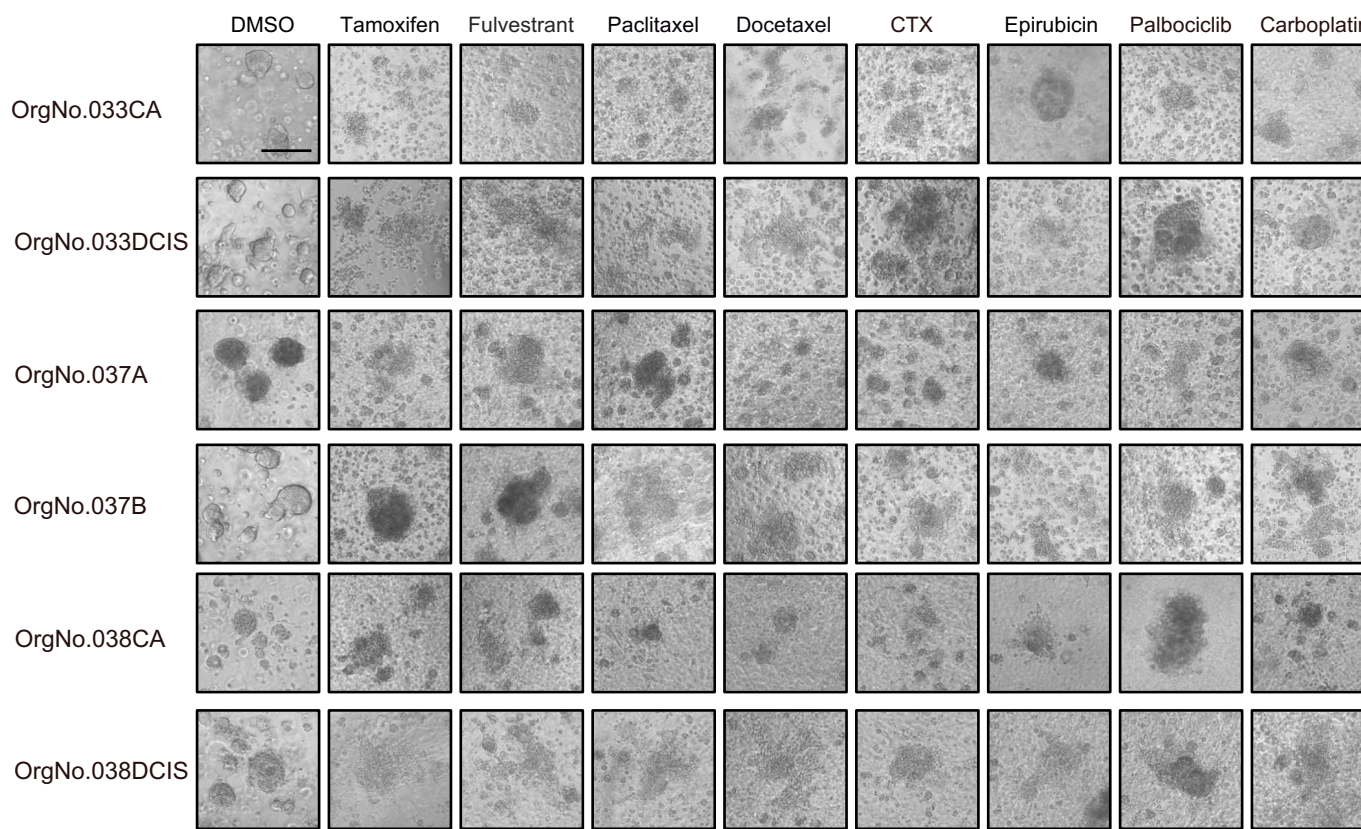

Supplement: Supplementary file 7 — Figure S7. Bright‐field microscopy images of in vitro drug responses of BC PDTOs to several clinically used drugs. [file CTM2-11-e380-s004.pdf]

Figure S8

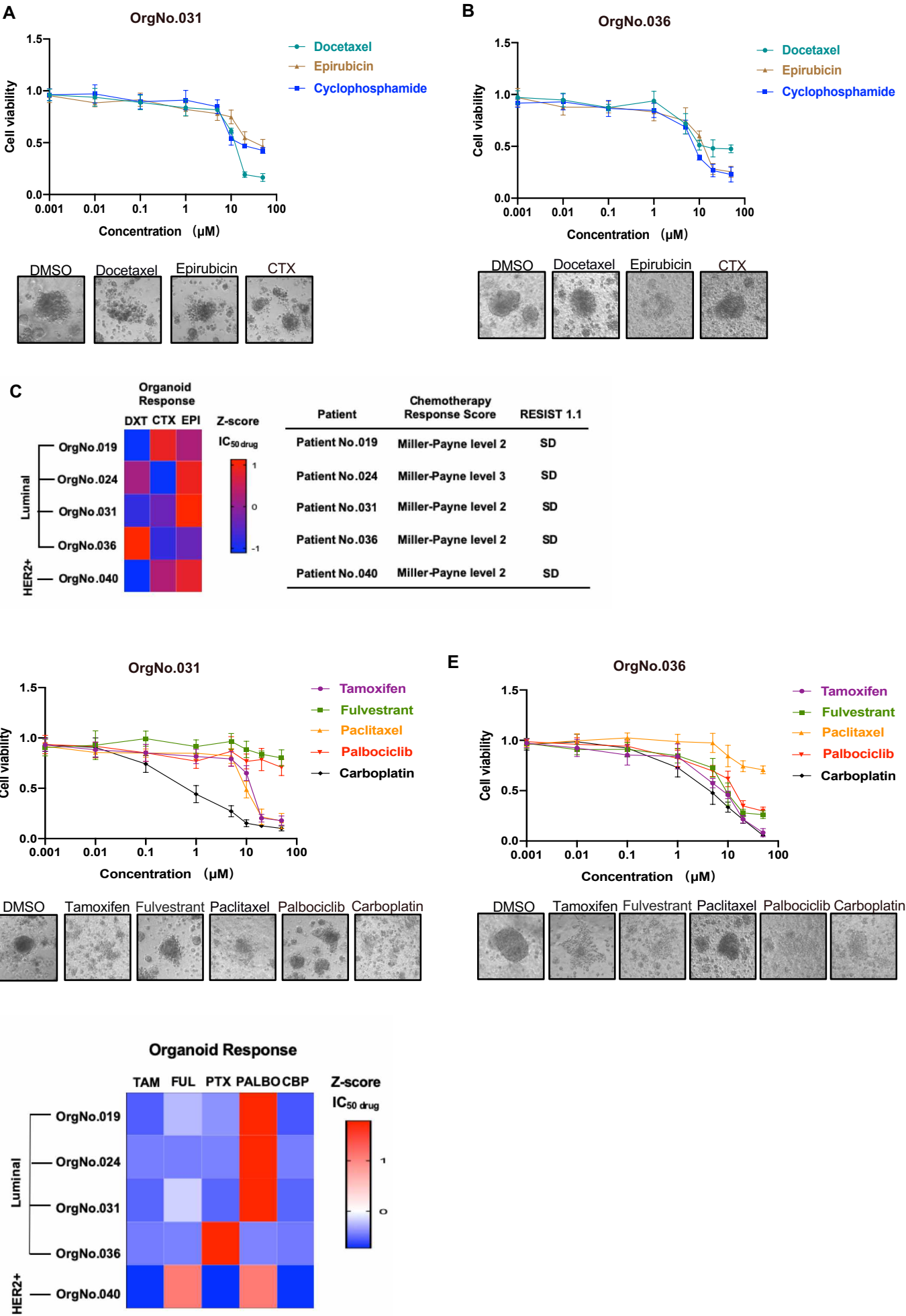

Supplement: Supplementary file 8 — Figure S8. PDTOs drug response correlates with clinical drug resistant response of original neoadjuvant BC patients and provides new treatment options for neoadjuvant BC patients. [file CTM2-11-e380-s001.pdf]

Figure S9

Bilateral breast cancer

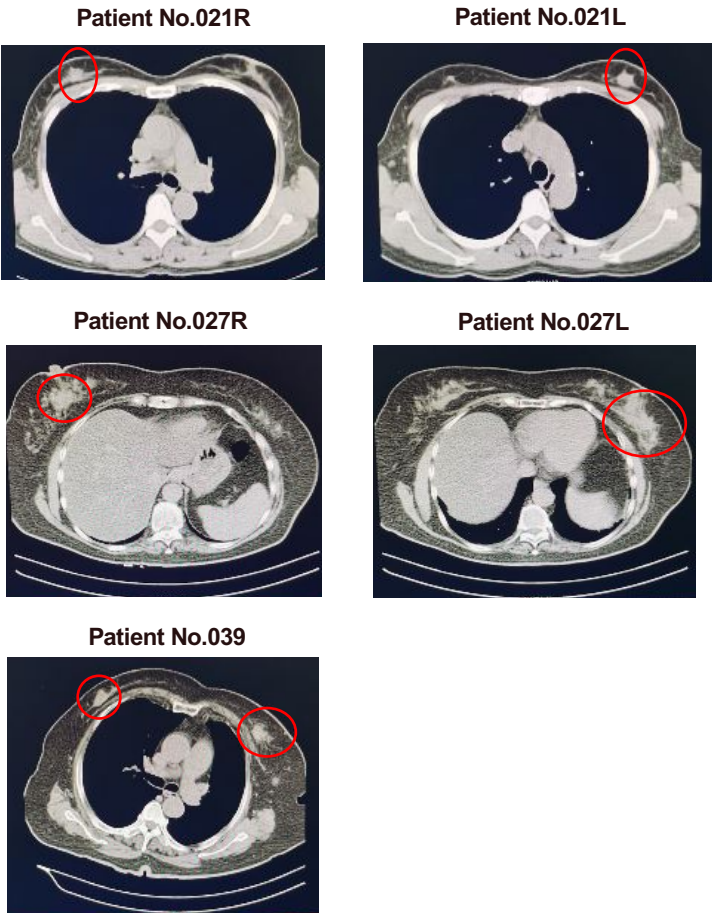

Multifocal breast cancer

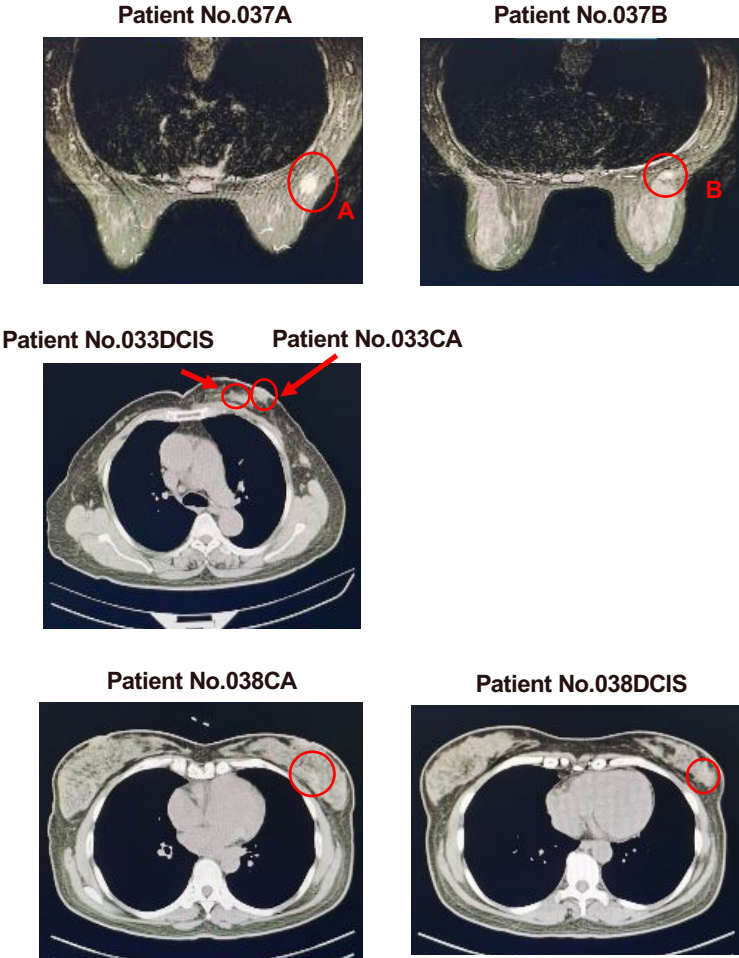

Supplement: Supplementary file 9 — Figure S9. Clinical imaging characteristics of bilateral BC patients and multifocal BC patients. [file CTM2-11-e380-s010.pdf]

Figure S10

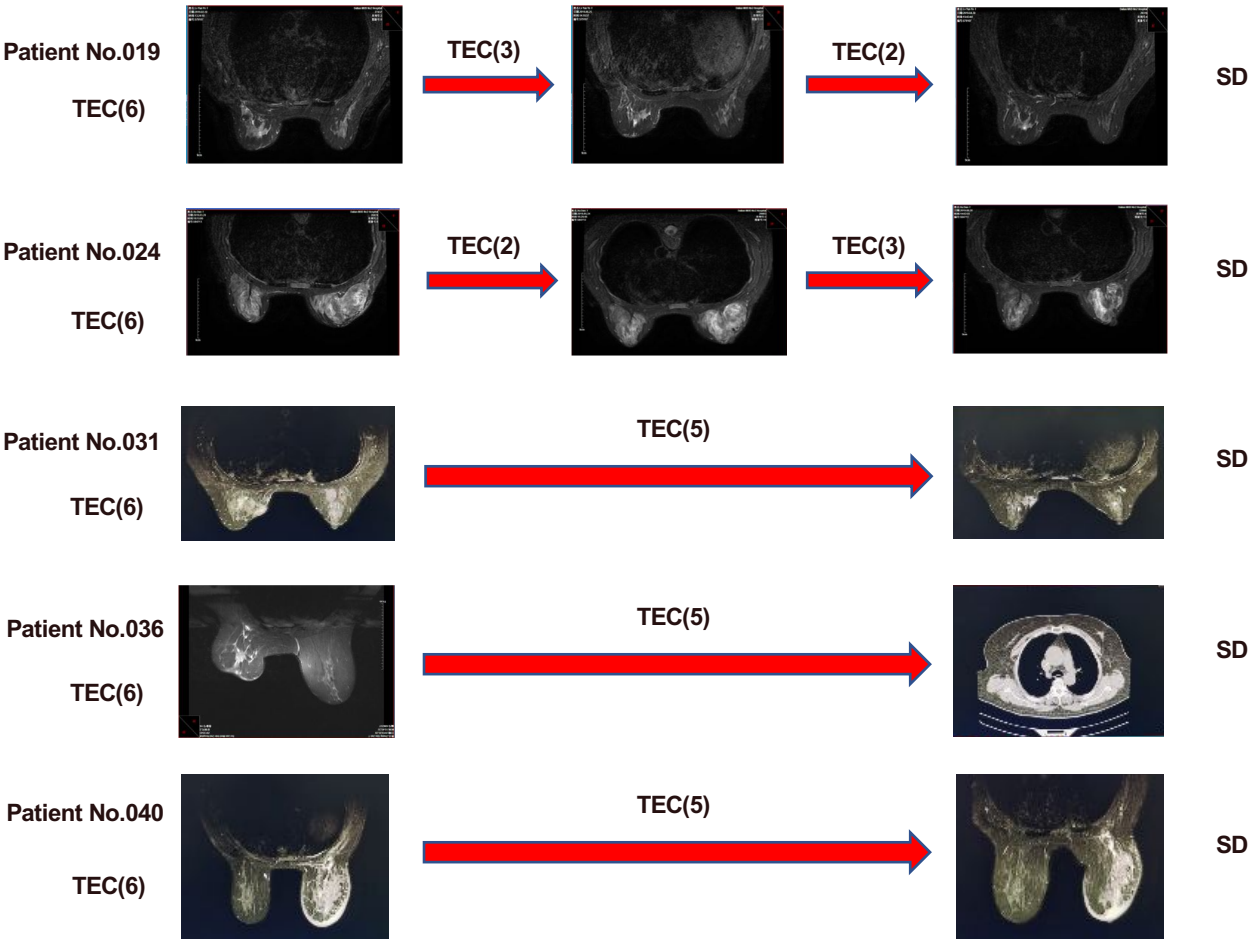

Supplement: Supplementary file 10 — Figure S10. Clinical imaging characteristics of tumor changes before and after neoadjuvant therapy in BC patients. [file CTM2-11-e380-s002.pdf]

## Figure S11

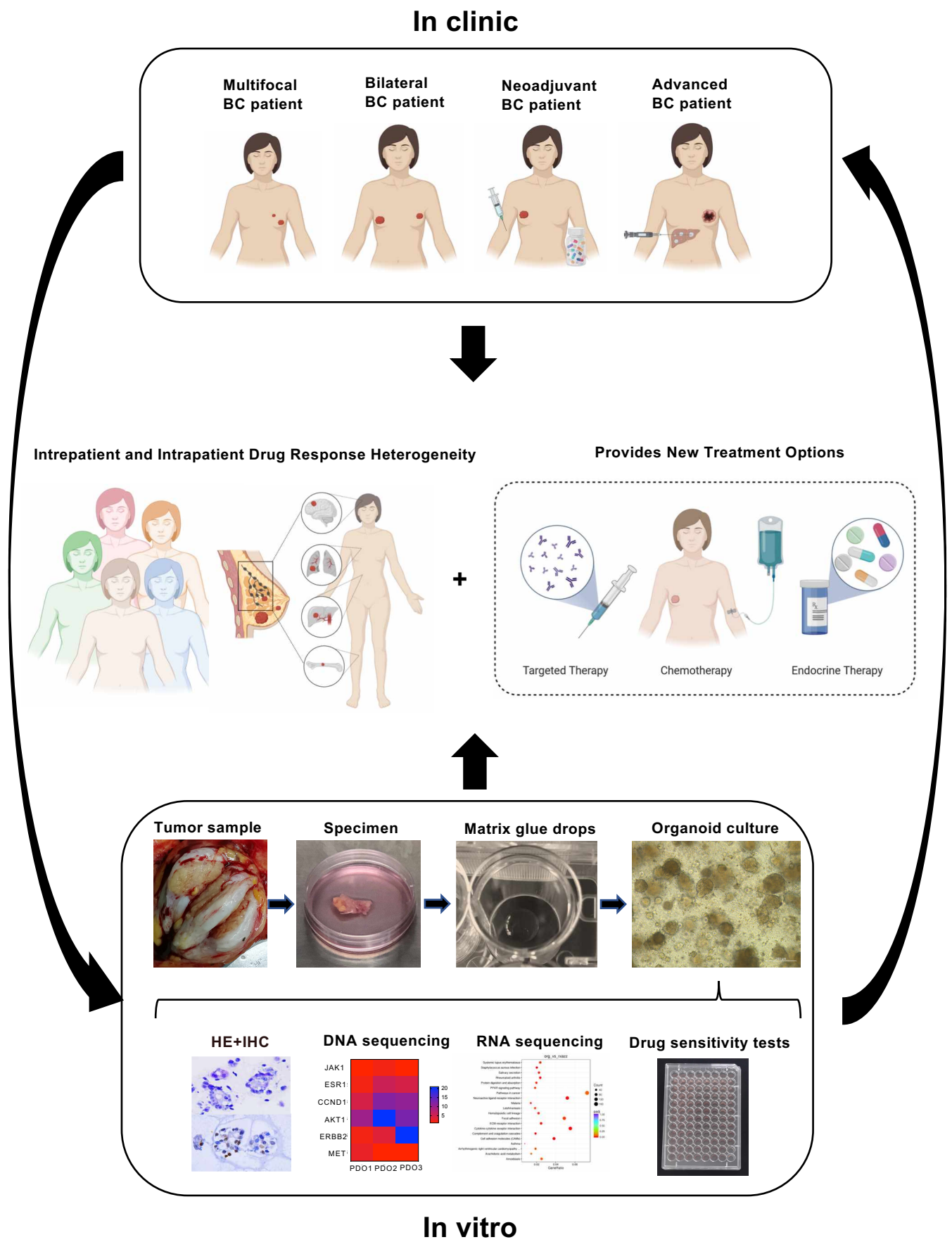

Supplement: Supplementary file 11 — Figure S11. PDTOs‐based co‐clinical trials in breast cancers. [file CTM2-11-e380-s008.pdf]
