# Supplementary material for: Optimizing individualized treatment strategy based on breast cancer organoid model
Source: Clin Transl Med. 2021 Mar 31;11(4):e380. doi: 10.1002/ctm2.380 (PMC8012563; doi:10.1002/ctm2.380)

Figure S6

A. Unilateral BC PDTOs

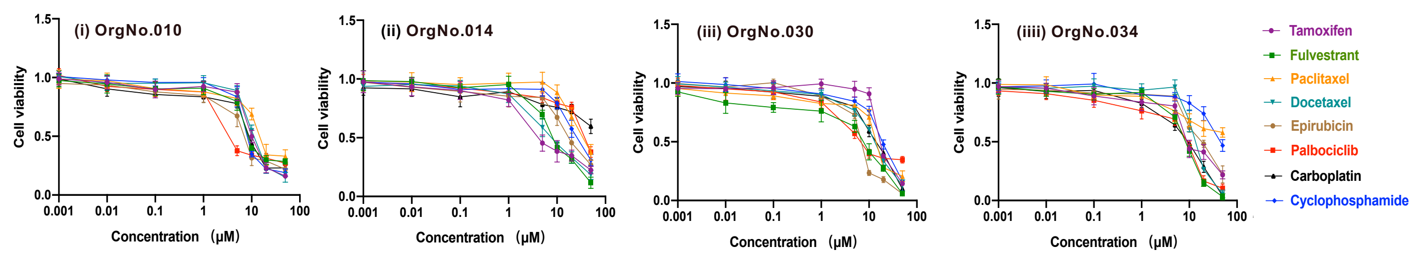

B. HER2-positive BC PDTOs

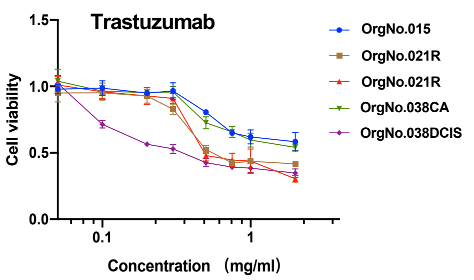

C

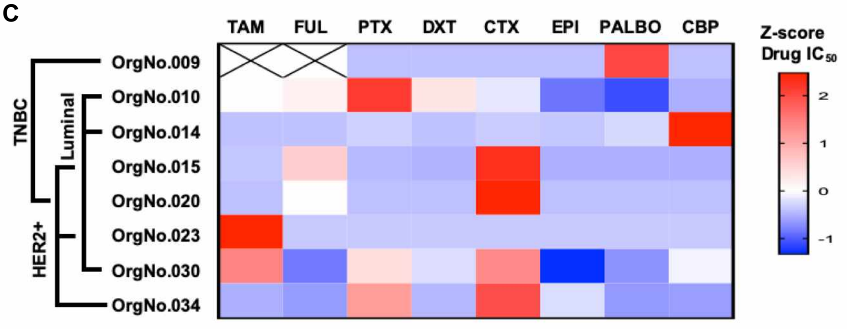

D. Multifocal BC PDTOs

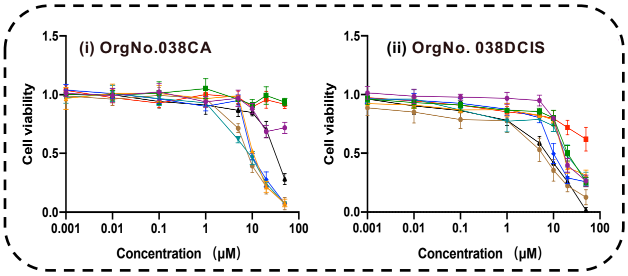

E. Bilateral BC PDTOs

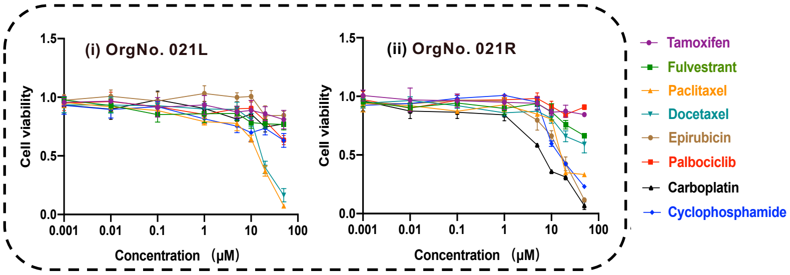

F

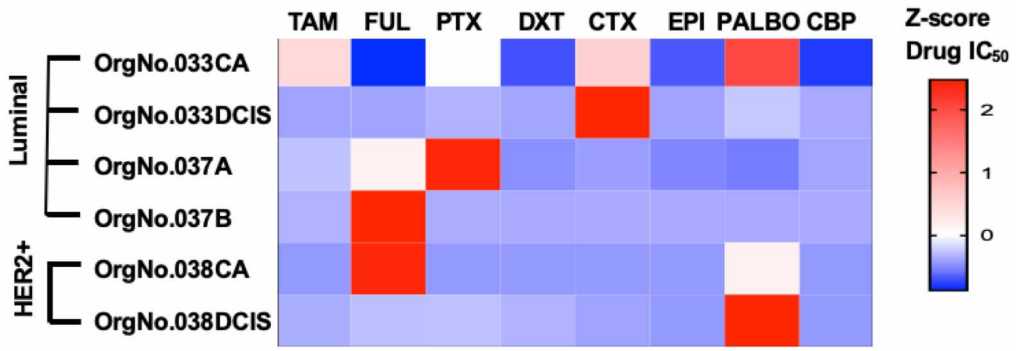

G

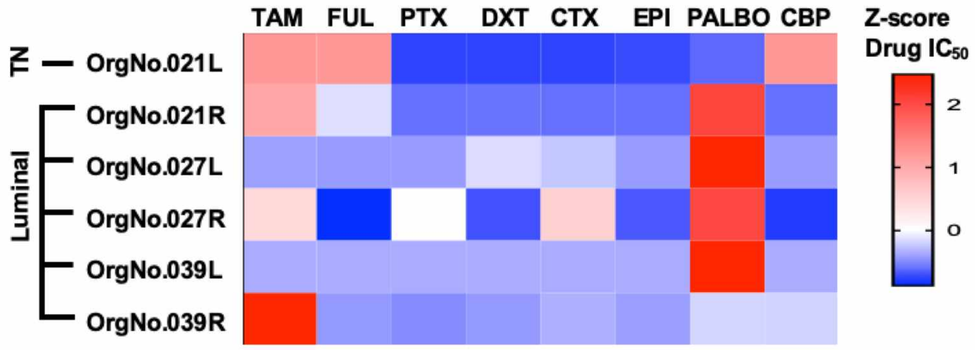

Supplement: Supplementary file 6 — Figure S6. Pharmacological characteristics of PDTOs derived from different BC patients. [file CTM2-11-e380-s006.pdf]
